# Supplementary material for: Evodiamine Exhibits Anti-Bladder Cancer Activity by Suppression of Glutathione Peroxidase 4 and Induction of Ferroptosis
Source: Int J Mol Sci. 2023 Mar 23;24(7):6021. doi: 10.3390/ijms24076021 (PMC10094601; doi:10.3390/ijms24076021)
Supplement: Supplementary file 1 [file ijms-24-06021-s001.zip › ijms-2213645-SI.pdf]

Supplementary information

Evodiamine exhibits anti-bladder cancer activity by suppression of glutathione peroxidase 4 and induction of ferroptosis

Che-Yuan Hu [1,2], Hung-Tsung Wu [3], Yan-Shen Shan [4,5], Chung-Teng Wang [6], Gia-Shing Shieh [7,8], Chao-Liang Wu [9,10*], Horng-Yih Ou [3,11*]

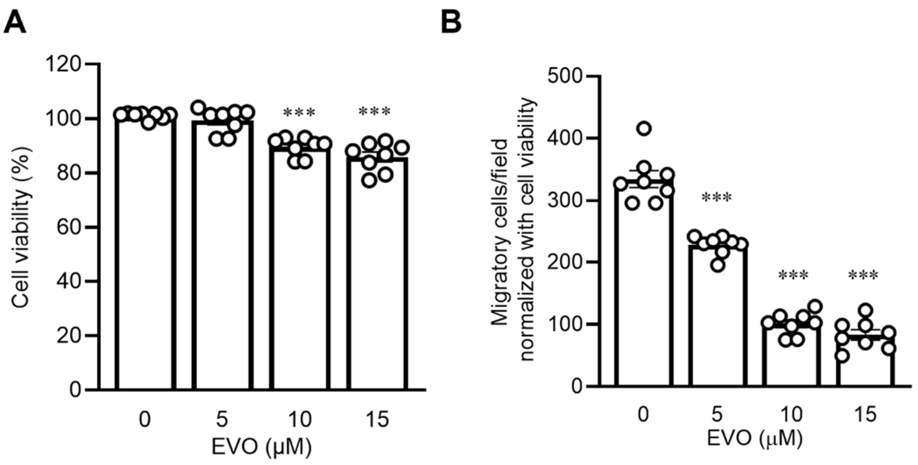

Figure S1. EVO at low concentrations inhibits cell migration in TCCSUP cells without evident cytotoxicity. (A, B) TCCSUP cells cultured in 10-cm culture dishes were treated with or without EVO for 24 h. The treated cells were then trypsinized, resuspended in the culture medium in the absence of EVO, reseeded at 1×10^4/well in 200 μl culture medium in 96-well plates (A) and in the upper chamber of the transwell (B), and cultured for an additional 24 h for assessing cell viability with the MTS assay (n = 8) (A) and cell migration with the transwell migration assay (B). Cells that migrated through the membrane to the lower surface were stained with Giemsa and quantified (n = 8) (B). To exclude the possibility that the cytotoxic effect of EVO may contribute to the reduced cell migration induced by EVO, the numbers of migratory cells were normalized with the percentage of cell viability shown in A.
